# Prevalence and Molecular Characterization of Chronic and Occult Hepatitis B Virus Infection Among Pregnant Women in St. Petersburg, Russia

**DOI:** 10.3390/ijms26189079

**Published:** 2025-09-18

**Authors:** Yulia V. Ostankova, Alexander N. Shchemelev, Elena N. Serikova, Marina B. Kusevitskaya, Maksim V. Sannikov, Olga A. Gorskaya, Valentina V. Basina, Natalia Yu. Shirshova, Ilya A. Mashkov, Elena B. Zueva, Diana E. Reingardt, Areg A. Totolian

**Affiliations:** 1 Saint Petersburg Pasteur Institute, 197101 St. Petersburg, Russia; shenna1@yandex.ru (Y.V.O.); genista.bio@gmail.com (E.N.S.); ezueva75@mail.ru (E.B.Z.); dianavalutite008@gmail.com (D.E.R.); totolian@spbraaci.ru (A.A.T.); 2 St. Petersburg State Institution of Health ‘City Clinical Hospital No 31’, 197110 St. Petersburg, Russia; mbkus@mail.ru; 3 Nikiforov Russian Center of Emergency and Radiation Medicine, EMERCOM of Russia, 197082 St. Petersburg, Russia; smakv@mail.ru; 4 D.O. Ott Research Institute of Obstetrics Gynecology and Reproduction, 199034 St. Petersburg, Russia; gorskaya70@rambler.ru; 5 Department of Adult Infectious Diseases and Epidemiology, Saint Petersburg State Pediatric Medical University, 194100 St. Petersburg, Russia; v.basina@mail.ru; 6 St. Petersburg State Budgetary Institution of Health ‘City Polyclinic No 32’, 197022 St. Petersburg, Russia; shirnat1@yandex.ru; 7 St. Petersburg State Budgetary Institution of Health ‘City Polyclinic No 99’, 194358 St. Petersburg, Russia; ilya.mashkov@list.ru

**Keywords:** hepatitis B virus, hepatitis B vaccine, pregnant women, occult hepatitis B virus infection, OBI, laboratory diagnostics

## Abstract

Hepatitis B virus (HBV) remains a major global health concern, as it is not only one of the most common hepatotropic viruses but also ranks as the seventh leading cause of mortality worldwide. The most significant routes of infection include vertical transmission (from mother to child before, during, or after birth, including transplacental infection) and horizontal transmission in early childhood through close household contact with infected parents. The aim of our study was to assess the prevalence of chronic and occult hepatitis B virus infection among pregnant women in St. Petersburg (Russia), including molecular characterization. We analyzed plasma samples from 1368 local pregnant women. ELISA screening for HBV markers included qualitative detection of HBsAg, anti-HBs IgG, and anti-HBcore IgG. HBV DNA was identified using highly sensitive nested PCR, followed by whole-genome sequencing for HBV DNA-positive cases. Our study evaluated the prevalence of serological and molecular HBV markers and their association with age, vaccination status, and number of pregnancies. Serological markers HBsAg, anti-HBs IgG, and anti-HBcore IgG were detected in 1.9%, 63.8%, and 12.9% of participants, respectively. HBV DNA was found in 4.7% of pregnant women, including 2.8% with occult HBV infection (OBI). We observed a positive correlation between anti-HBcore IgG and age, but an inverse correlation with anti-HBs IgG; an inverse correlation between anti-HBcore IgG and vaccination status, while anti-HBs IgG showed a positive correlation; and a positive correlation between HBsAg, anti-HBcore IgG, and HBV DNA with the number of pregnancies. We also analyzed the prevalence of clinically significant mutations, including drug resistance mutations, escape mutations (affecting diagnostic detection and vaccine efficacy), and mutations associated with disease progression. The detection of HBsAg-negative HBV infection was linked to circulating viral variants carrying escape mutations, which evade HBsAg detection in diagnostic assays and neutralization by vaccine-induced antibodies. The predominance of HBV isolates in pregnant women harboring dual-threat mutations (those causing diagnostic failure via HBsAg negativity, reduced vaccine/immunoglobulin efficacy, viral reactivation, disease progression) poses a significant public health risk and warrants further investigation.

## 1. Introduction

Bloodborne viral infections remain a global public health challenge, with transmission occurring through exposure to blood and other bodily fluids [1]. Among these, hepatitis B virus (HBV) is of particular concern worldwide, ranking as both one of the most prevalent hepatotropic viruses and the seventh leading cause of global mortality. The failure to seroconvert (HBsAg/anti-HBs IgG) within six months of infection defines chronic HBV (CHB). When infection occurs early (before age five), the vast majority of cases progress to chronic hepatitis B [2]. Early HBV acquisition is also a major risk factor for liver cirrhosis and hepatocellular carcinoma (HCC) [3]. Consequently, vertical transmission (mother-to-child before, during, or after birth, including transplacental exposure) and horizontal transmission in early childhood through close contact with infected caregivers are considered critical routes of infection, with the first month of life being the most vulnerable period [4]. Without intervention, mother-to-child transmission rates exceed 31% [5].

HBV prevalence is classified by HBsAg seroprevalence: low endemicity <2%, intermediate 2–7%, or high ≥8% [6]. Current estimates indicate 290–360 million people live with CHB worldwide [7]. This high prevalence stems partly from HBV’s exceptionally low infectious dose [8]. 

However, HBsAg negativity does not rule out infection. A natural form of CHB is HBsAg-negative or occult hepatitis B infection (OBI), wherein HBV DNA persists in hepatocytes despite undetectable serum HBsAg, regardless of detectable viremia [9]. Viral replication and gene expression may be suppressed to levels where blood viral loads fall below detection limits of standard assays, yet the virus persists [9]. OBI prevalence correlates with regional HBsAg rates and depends on: overall HBV burden, vaccination programs, and population risk factors. However, comparative estimates are challenging due to methodological differences in HBV detection and heterogeneity in studied cohorts [10,11,12,13].

The clinical significance of OBI remains debated. While low/undetectable viremia and HBsAg clearance represent the therapeutic goal for CHB, OBI is paradoxically linked to: accelerated liver disease progression in HCV coinfection or other liver pathologies [13,14]; increased HCC risk even in isolated OBI without comorbidities [15]; and reactivation risk (∼40%) during immunosuppression (e.g., cancer therapy) [16,17].

Currently, HBV is classified into ten genotypes (A-J) with more than 8% divergence in their whole-genome nucleotide sequences. Additionally, genotypes A, B, C, D, F, and H contain subgenotypes that differ by 4–7.5% from each other [18]. Most geographical regions are predominantly dominated by 2–3 subgenotypes of one or two major viral genotypes, along with several minor ones including isolated imported cases from other regions [18,19,20]. HBV’s remarkable genetic variability enables the virus to respond to endogenous and exogenous selective pressures through continuous modulation of its genome structure. Consequently, genotype alone does not determine disease progression; naturally occurring and selection-driven viral mutations also contribute significantly. During prolonged infection and under various pressures, the virus can evolve to evade therapeutic interventions, preventive measures, and diagnostic detection [21].

Clinically significant mutations primarily include drug resistance mutations (DRMs) occurring in the P gene. The emergence and selection of nucleos(t)ide analog resistance mutations in the P region provide a survival advantage to the virus and represent a major obstacle to successful treatment [22]. 

Mutations enabling immune escape, known as ‘escape mutations’ (EMs), play a crucial role, predominantly occurring in the N-terminal region and major hydrophilic region (MHR) of the S domain. These mutations facilitate viral evasion from both serological detection and immune responses [23]. This phenomenon occurs because the dominant epitopes of HBsAg (the primary targets for B-cell neutralizing responses) are located within the “a” determinant of the MHR. Mutations in these regions induce conformational changes in the epitope that reduce binding affinity between HBsAg and anti-HBs antibodies [24]. One proposed mechanism suggests that amino acid substitutions in this region (such as G145R, Y100C) can alter the hydrophilicity, electrical charge, and/or acidity of HBsAg. This may affect HBV antigenicity, leading to either immune evasion or false-negative HBsAg screening results [23]. The genomic overlap between the S gene and the reverse transcriptase (RT)-encoding fragment of the *P* gene means that EMs in the MHR can emerge following DRMs in the RT region, and vice versa [25]. For instance, a significant proportion of patients carrying the rtA181T mutation also harbor the nonsense mutation sW172*, which results in truncated pre-S/S proteins [22].

The core protein’s pivotal role in the HBV life cycle explains why clinically significant mutations in the precore/core region are associated with disease progression. With rare exceptions (deletions), most genomic mutations in this region represent point substitutions primarily linked to reduced HBeAg levels and/or decreased viral load. Notably, core region mutations predominantly cluster within immunologically active domains (MHC class I + II epitopes), thereby potentially influencing disease development [26]. The basal core promoter (BCP), which regulates production of both precore and pregenomic RNA, also plays a decisive role in viral replication. Mutations in this region may similarly contribute to disease progression [27,28,29]. Established associations exist between core region mutations, preS deletions, and C-terminal truncations of envelope proteins with progressive liver disease and HCC development. Furthermore, mutations suppressing HBeAg expression have been linked to acute liver failure [22].

Assessing the prevalence of chronic HBV markers, including OBI, and conducting dynamic surveillance of circulating viral variants in key population groups that reflect broader epidemiological trends and may contribute to pathogen transmission represents a critical objective in contemporary virology. This importance stems from the fact that molecular-genetic variability in the virus may correlate with spatiotemporal patterns, evolving over time through geographic spread across different regions, risk groups, and key populations, as well as through shifts in transmission routes [30].

Screening pregnant women for HBV carries particular significance. Globally, approximately 75 million women of reproductive age live with chronic HBV infection, representing about 25.3% of cases [5]. Given the WHO’s goal to eliminate HBV as a public health threat by 2030, preventing mother-to-child transmission through universal infant vaccination and hepatitis B immunoglobulin administration to newborns of infected women remains a prevention priority [31]. Focusing on infected women and providing prophylactic treatment to those with high viral loads may prove an effective strategy for reducing vertical transmission and improving control of this socially significant disease [32,33,34]. However, our understanding of chronic HBV progression in pregnant women remains limited. Although OBI typically presents with low viral loads, the potential for vertical transmission or postpartum horizontal transmission through maternal-neonatal contact cannot be ruled out. This risk becomes particularly concerning when dealing with escape mutant strains, as these variants may persist despite vaccination efforts [35,36]. Underdiagnosis of HBV in risk groups and populations with potential transmission (including pregnant women) continues to pose a major obstacle to eliminating hepatitis B as a public health threat [1].

Pregnant women serve as sentinel populations that mirror community-wide epidemiological patterns, effectively representing the infection profile of sexually mature heterosexual adults within a given geographic region. Consequently, detecting chronic HBV (including OBI) in pregnant women, along with characterizing circulating HBV genotypes and mutations in these groups, becomes essential. Such work is valuable in: evaluating epidemic trends, informing timely therapeutic decisions; optimizing immunoprophylactic strategies; and planning effective pathogen containment measures. The aim of our study was to assess the prevalence of chronic and occult hepatitis B virus infection among pregnant women (St. Petersburg, Russia), including molecular characterization

## 2. Results

### 2.1. Hepatitis B Vaccination Status

In the study group, 1088 individuals (79.5%; 95% CI: 77.3–81.6%) had been vaccinated against HBV. A decreasing trend in vaccination rates was observed with increasing age (Table 1).

No significant differences were found in vaccination rates between the 18–21, 22–25, and 26–31 year-old groups. The proportion of vaccinated women in the combined 18–31 age group was 86.7% (95% CI: 84.4–88.8%). Significant differences in HBV vaccination rates were observed between: the 18–31 and 32–36 year-old groups (χ^2^ = 64.7, *p* < 0.0001, df = 1); and the 32–36 and 37–46 year-old groups (χ^2^ = 6.3, *p* = 0.0118, df = 1).

### 2.2. Prevalence and Distribution of HBV Serological Markers in the Study Group

When assessing the overall prevalence of serological markers among pregnant women, only 363 women showed no HBV-associated markers, accounting for 26.5% (95% CI: 24.2–29.0%). Detections were as follows: HBsAg was detected in 26 individuals (1.9%, 95% CI: 1.3–2.8%); anti-HBs IgG antibodies were detected in 873 women (63.8%, 95% CI: 61.2–66.4%); and anti-HBcore IgG Abs were detected in 177 pregnant women (12.9%, 95% CI: 11.2–14.8%). The prevalence of serological markers across different age groups is presented in Table 2.

Comparative analysis of serological marker prevalence across age groups revealed significant differences in anti-HBs IgG antibody distribution: χ^2^ = 33.4, *p* < 0.0001, df = 4. In sequential pairwise comparisons, no differences were found in the frequency of this marker between the 18–21, 22–25, and 26–31 year-old groups. The proportion of women with anti-HBs IgG in the 18–31 year-old group was 68.5% (95% CI: 65.4–71.4%). Similarly, no differences were observed between the 32–36 and 37–46 year-old groups. The anti-HBs IgG prevalence in the 32–46 year-old group was 52.9% (95% CI: 47.9–57.8%). Significant differences in anti-HBs IgG frequency were demonstrated between the 18–31 and 32–46 year-old groups (χ^2^ = 29.4, *p* < 0.0001, df = 1).

Significant differences in anti-HBcore IgG antibody prevalence were observed across age groups: χ^2^ = 69.4, *p* < 0.0001, df = 4. Sequential pairwise comparisons showed no differences in this marker’s frequency between the 18–21, 22–25, and 26–31 year-old groups, nor between the 32–36 and 37–46 year-old groups. The proportions of women with anti-HBcore IgG in the 18–31 and 32–46 year-old groups were 8.1% (95% CI: 6.5–10.1%) and 24.2% (95% CI: 20.1–28.6%), respectively. The anti-HBcore IgG frequency was significantly lower in the 18–31 year-old group compared to the 32–46 year-old group (χ^2^ = 63.9, *p* < 0.0001, df = 1). No differences in HBsAg frequency were found between age groups. The prevalence of the aforementioned serological markers was analyzed relative to the number of pregnancies (Table 3).

No differences were found in the prevalence of HBsAg, anti-HBs IgG antibodies, or anti-HBcore IgG antibodies between groups with first and second pregnancies. The occurrence of these markers in the combined group was 1.4% (95% CI: 0.9–2.3%), 64.9% (95% CI: 62.2–67.6%), and 9.9% (95% CI: 8.3–11.7%), respectively. The frequency of HBsAg was significantly higher among women with third or fourth pregnancies compared to those with first or second pregnancies (χ^2^ = 13.1, *p* < 0.0001, df = 1). The likelihood of marker presence among women with third/fourth pregnancies was nearly four times higher than among women with first/second pregnancies (OR = 4.8, 95% CI: 2.1–11.4%). The occurrence of anti-HBcore IgG antibodies was also significantly higher in the group including women with third, fourth, or fifth pregnancies (44.3%; 95% CI: 35.3–53.5%) compared to women with first/second pregnancies (χ^2^ = 113.6, *p* < 0.0001, df = 1, OR = 7.25, 95% CI: 4.8–10.8%). Conversely, the prevalence of anti-HBs IgG antibodies was significantly higher in the group with first/second pregnancies than in the group with third/fourth pregnancies (χ^2^ = 6.4, *p* = 0.0113, df = 1). The distribution of serological markers in the study group of pregnant women is presented in Table 4.

The distribution of serological markers was analyzed according to the number of pregnancies (Table 5).

It should be noted that while samples with isolated anti-HBcore IgG antibodies, as well as those with combined anti-HBs IgG and anti-HBc IgG, showed approximately equal representation of women with first, second, and third pregnancies, the majority of those positive for isolated anti-HBs IgG were women with first and second pregnancies.

### 2.3. Prevalence of HBV DNA in the Study Group

Among the examined pregnant women, HBV DNA was detected in 64 individuals, representing 4.7% (95% CI: 3.6–5.9%). The occurrence of HBV DNA across different age groups is presented in Table 6.

In sequential pairwise comparisons, no significant differences in HBV DNA frequency were found between age groups. However, HBV DNA occurrence was significantly higher in the 32–36 year-old group compared to the 22–25 year-old group (Fisher’s exact test *p* = 0.0313, OR = 2.6, 95% CI: 1.1–6.2%). The prevalence of HBV DNA according to pregnancy number is shown in Table 7.

Significant differences in HBV DNA occurrence were found depending on pregnancy number (excluding the single case with five pregnancies): χ^2^ = 61.7, *p* < 0.0001, df = 3. Sequential pairwise comparisons revealed that HBV DNA detection frequency was significantly higher among women with two pregnancies compared to those with one pregnancy (χ^2^ = 4.6, *p* = 0.0312, df = 1, OR = 2.04, 95% CI: 1.1–3.8%). Furthermore, women with three pregnancies showed higher HBV DNA prevalence than those with two pregnancies (Fisher’s exact test *p* = 0.0006, OR = 3.4, 95% CI: 1.8–6.4%).

Notably, only 26 women had detectable HBV DNA together with HBsAg (1.9%, 95% CI: 1.3–2.8% of the total group), while 38 individuals (2.8%, 95% CI: 1.97–3.8%) were HBsAg-negative, representing OBI cases (Table 8).

No significant differences were found in chronic HBV versus OBI frequencies across age groups. Table 9 shows the distribution of serological markers among HBV DNA-positive women.

Interestingly, among HBV DNA-positive individuals, only OBI cases showed either isolated anti-HBc IgG or combined anti-HBs IgG and anti-HBc IgG. Moreover, two OBI cases had no detectable serological markers, while one showed isolated anti-HBs IgG antibodies.

### 2.4. Association Between HBV Vaccination, Age, Number of Pregnancies, and Analyzed Markers

**Figure 1:**
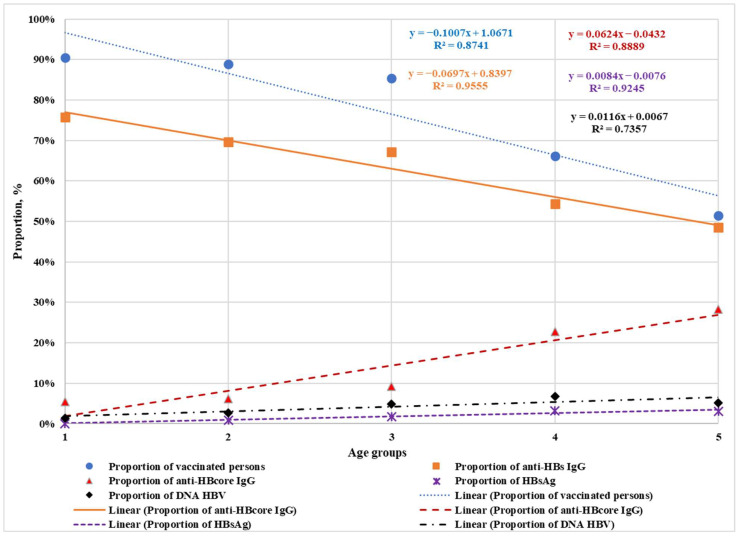
Proportions of vaccinated individuals, those with anti-HBs IgG, anti-HBc IgG, HBsAg, and HBV DNA across age groups of pregnant women. The numbers indicate age groups: 1—18–21 years, 2—22–25 years, 3—26–31 years, 4—32–36 years, 5—37–46 years.

Negative linear trends were identified showing decreasing proportions of vaccinated individuals and those with anti-HBs IgG with increasing age across age groups. Positive linear trends were observed reflecting increasing proportions of individuals with anti-HBcore IgG, HBsAg, and HBV DNA with advancing age (Figure 1).

Spearman’s rank correlation coefficients were calculated for all analyzed parameters relative to age. With *p* < 0.05, np = 29, and critical Spearman’s rank correlation coefficient = 0.37, the following correlations were found: negative correlation between proportion of vaccinated individuals and age (rs = −0.79); negative correlation between proportion of anti-HBs IgG-positive individuals and age (rs = −0.51); and positive correlation between proportion of anti-HBc IgG-positive individuals and age (rs = 0.62). No correlation was found between proportions of HBsAg-positive or HBV DNA-positive individuals and age.

Spearman’s correlation coefficients were also calculated for all markers relative to vaccination status. With *p* < 0.05, np = 29, and critical Spearman’s rank correlation coefficient = 0.37: positive correlation existed between proportion of anti-HBs IgG-positive individuals and vaccination rate (rs = 0.77); and negative correlation between proportion of anti-HBc IgG-positive individuals and vaccination rate (rs = −0.6). No correlation was found between proportions of HBsAg-positive or HBV DNA-positive individuals and vaccination rate.

**Figure 2:**
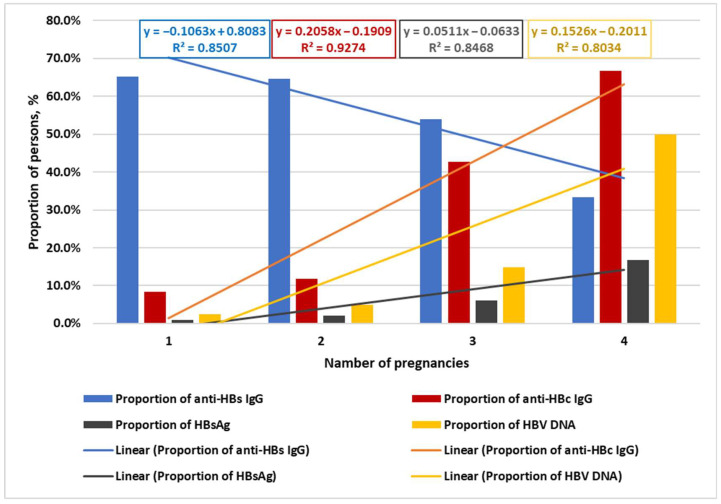
Proportions of individuals with anti-HBs IgG, anti-HBc IgG, HBsAg, and HBV DNA across groups with different numbers of pregnancies (1 to 4).

A negative linear trend was identified showing decreasing proportion of anti-HBs IgG-positive individuals with increasing number of pregnancies. Positive linear trends were observed reflecting increasing proportions of individuals with anti-HBcore IgG, HBsAg, and HBV DNA with greater number of pregnancies (Figure 2).

**Figure 3:**
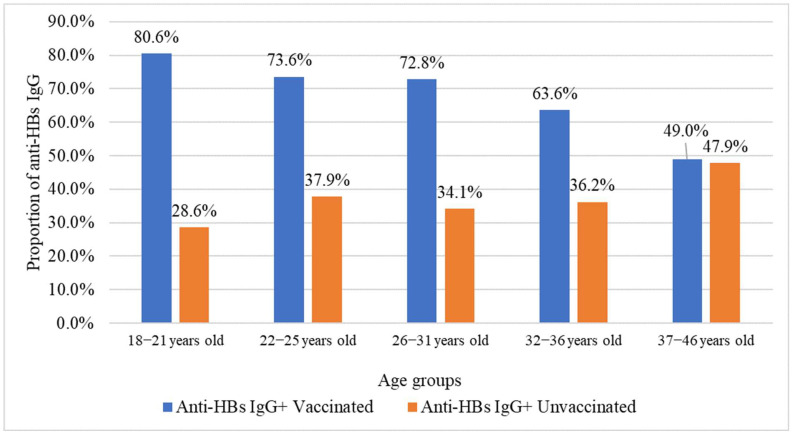
Frequency of anti-HBs IgG among pregnant women by vaccination status.

The prevalence of anti-HBs IgG and anti-HBcore IgG was evaluated according to vaccination status. The frequency of anti-HBs IgG among vaccinated and unvaccinated individuals was 70.6% (95% CI: 67.8–73.3%) and 37.5% (95% CI: 31.8–43.5%), respectively. Age-stratified analysis revealed significant differences in anti-HBs IgG frequency between vaccinated and unvaccinated pregnant women (*p* < 0.05), with significantly higher anti-HBs IgG prevalence among vaccinated individuals across all age groups, except 37–46 years (Figure 3).

**Figure 4:**
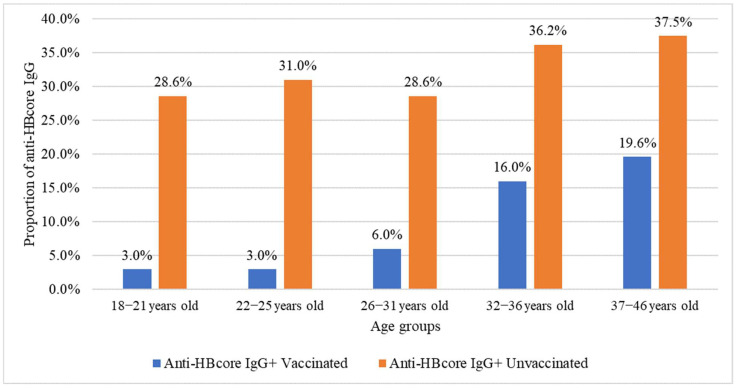
Frequency of anti-HBcore IgG among pregnant women by vaccination status.

The frequencies of anti-HBcore IgG among vaccinated and unvaccinated individuals were 13.2% (95% CI: 11.3–15.4%) and 33.2% (95% CI: 27.7–39.1%), respectively. Age-stratified analysis showed significant differences in anti-HBcore IgG frequency between vaccinated and unvaccinated pregnant women (*p* < 0.05), with significantly higher anti-HBcore IgG prevalence among unvaccinated individuals across all age groups, except 37–46 years (Figure 4, Table 10).

Significant differences were noted in the distribution of vaccinated versus unvaccinated individuals among HBV DNA-positive cases based on HBsAg status.

### 2.5. HBV Genotyping

**Figure 5:**
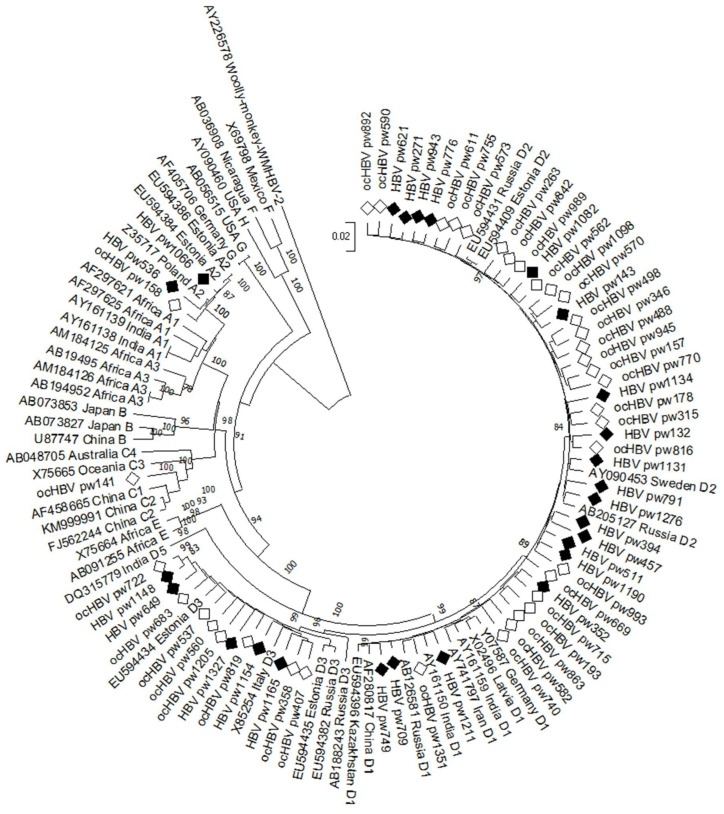
Phylogenetic analysis of HBV whole-genome nucleotide sequences isolated from pregnant women. Reference sequences available in GenBank were used for comparison. Reference sequences are designated with GenBank codes indicating genotype and region of sample origin. The Woolly Monkey HBV nucleotide sequence AY226578 was used as the outer group. The samples studied in this work are indicated by black diamonds (HBsAg+) and white diamonds (HbsAg−). Bootstrap values ≥ 70.

The whole-genome nucleotide sequences of identified HBV isolates have been deposited in GenBank under accession numbers PQ601122-PQ601185. Phylogenetic analysis was performed on the obtained nucleotide sequences (Figure 5).

Comprehensive genotyping and subtyping analysis incorporating phylogenetic evaluation and web-based resources revealed predominant circulation of genotype D (n = 60, 93.8%, 95% CI: 84.8–98.3%) compared to genotypes A (n = 3, 4.7%, 95% CI: 1.0–13.1%) and C (n = 1, 1.5%, 95% CI: 0.0–8.4%). Among genotype D cases, subgenotypes D1 (26.67%), D2 (51.67%), and D3 (21.67%) were identified. Genotype A isolates belonged to subgenotype A2, while genotype C corresponded to subgenotype C1. Thus, the subgenotype distribution among pregnant women was as follows: D2—48.4%, D1—25.0%, D3—20.3%, A2—4.7%, and C1—1.5%.

### 2.6. Identification of Clinically Significant HBV Mutations

Multiple amino acid substitutions were identified in the RT, SHB, MHB, LHB, and core regions of HBsAg-negative and HBsAg-positive isolates from genotypes D and C. Genotype A isolates also exhibited multiple substitutions across all specified regions, except for sample HBV_pw1165, which showed no core region mutations. Precore region mutations were detected in 25 samples (39.1%, 95% CI: 27.1–52.1%).

### 2.7. Analysis of RT Region Mutations

Drug resistance mutations in the HBV reverse transcriptase region were identified in 3 cases (4.69%, 95% CI: 0.98–13.09%). All three cases featured the L180M+M204V mutation combination, with two cases additionally showing T184A. These mutations confer resistance to lamivudine, entecavir, and telbivudine. Notably, all DRM cases occurred in women with OBI. One additional case showed the L80F substitution (not previously classified as DRM) at a position associated with lamivudine/telbivudine resistance. All three genotype A isolates (4.69%, 95% CI: 0.98–13.09%) carried the L217R mutation linked to reduced adefovir sensitivity. The M129L substitution was detected in six cases (9.38%, 95% CI: 3.52–19.30%), while the HCC-associated M309K mutation appeared in 12 cases (18.75%, 95% CI: 10.08–30.46%).

### 2.8. Analysis of Mutations in the MHR of the HBV Genome

The prevalence of amino acid substitutions in the MHR was evaluated. The results are shown in Table 11 and Table 12.

Additionally, samples from HBV-vaccinated individuals with OBI and anti-HBs IgG antibodies were analyzed. The results are shown in Table 13.

### 2.9. Analysis of Mutations in the Precore/Core Region of the HBV Genome

Amino acid variability in the precore region was identified in 25 individuals (39.1%, 95% CI: 27.1–52.1%), with 9 polymorphic sites showing amino acid substitutions. Notably, mutations in this region were only detected in genotype D isolates. No significant differences were found in precore mutation frequency between HBsAg-positive and HBsAg-negative samples. The frequencies of the identified mutations are presented in Table 14.

In the core region, variability was detected in 63 patients (98.4%, 95% CI: 91.6–99.96%), with 70 polymorphic sites showing amino acid substitutions. The frequencies of mutations identified in the core region are presented in Table 15.

In the BCP region, the double mutation A1762T/G1764A was detected in 12 samples (18.8%, 95% CI: 10.1–30.5%), found in genotype D and C isolates.

## 3. Discussion

The current epidemiological situation in Russia regarding viral hepatitis B is characterized by a decline in reported cases of acute hepatitis B (AHB), but persistently high rates of CHB. St. Petersburg is among the Russian regions where both AHB and CHB incidence exceed the national average [37]. Given that epidemic activity of viral hepatitis is primarily driven by chronic infections, accurate epidemiological forecasting and effective prevention programs require comprehensive surveillance of all CHB cases, including OBI, particularly in key populations such as pregnant women.

Mass HBV vaccination over the past two decades represents a major public health achievement, significantly reducing hepatitis B prevalence among children. In Russia, vaccination against viral hepatitis B is carried out according to the 0-3-6 schedule (1st dose—at the start of vaccination, 2nd dose—3 months after the 1st vaccination, 3rd dose—6 months after the start of immunization) for newborns and all children not belonging to risk groups [38]. Current timely HBV vaccination coverage stands at 96.9%. However, hepatitis B predominantly affects adults, despite a 93.08% vaccination coverage rate [39].

In our study cohort of pregnant women, the vaccination rate was 79.5%, markedly lower than the reported adult vaccination statistics. We hypothesize that this discrepancy may stem from two factors. First, the possibility of artificially inflated vaccination rates in regional statistical reports cannot be ruled out. Second, study participants themselves may not remember whether they received HBV vaccination. While vaccination status can be verified through clinical records from primary care facilities, confirming non-vaccination remains problematic due to the absence of a nationwide automated vaccination monitoring system. In practice, individuals vaccinated outside their registered medical facility who fail to provide documentation are classified as unvaccinated.

Indirect support for the second hypothesis (poor recall) comes from the 37.5% anti-HBs IgG seropositivity rate among unvaccinated pregnant women, which is unlikely to result solely from natural infection. Conversely, all groups included unvaccinated individuals without anti-HBs IgG, indicating deliberate vaccine refusal despite pregnancy planning. 

Of particular note are the significant differences in HBV vaccination rates between the 18–31 age group (86.7%), the 32–36 group (66.2%), and the 37–46 group (51.5%). Moreover, the study population featured a negative correlation between vaccination rates and increasing age. These disparities likely reflect the fact that HBV vaccination was only introduced into Russia’s national immunization schedule in 1997. Adolescent vaccination at age 13 was incorporated into the national immunization program in 2001, achieving 96.5% coverage among children and 10–40% among high-risk adults, including occupational groups. Mass vaccination of the Russian population began in 2006 under the National Health Project, which included supplemental vaccination of adults aged 18–55 [40]. Thus, the declining vaccination rates with advancing age in our study cohort mirror the phased implementation of HBV vaccination nationwide.

While monitoring documented vaccination status is important, its value remains limited as it fails to reflect the key outcome: actual population immunity across different age and social groups. Under conditions of heterogeneous vaccine coverage, serological monitoring (as a component of epidemiological surveillance systems for immunization programs) becomes particularly valuable for tracking both population and individual immunity [41]. Our study revealed significant differences in anti-HBs IgG prevalence between the 18–31 (68.5%) and 32–46 (52.9%) age groups, along with a negative linear correlation between antibody levels and age. This likely stems from two factors: first, the age-related vaccination rate decline discussed above; and second, the gradual waning of post-vaccination antibody levels. Despite decades of HBV vaccination experience, no consensus exists regarding the duration of protective immunity.

It should be noted that the frequencies of HBsAg (1.9%) and anti-HBcore IgG (12.9%) detected in this study slightly exceed the regional prevalence of these markers: 1.3% and 11.3%, respectively. In St. Petersburg, these markers occur at significantly lower frequencies among blood donors (0.43% and 7.48%) and healthcare workers (0.58% and 10.53%), but are substantially higher among incarcerated individuals (3.2% and 37.68%) [42,43,44]. The highest prevalence of HBsAg and anti-HBs IgG in our study was observed in the 32–46 age group. During the study period (2021–2023), the peak prevalence of chronic HBV in the region occurred among 30–49 year-olds and the 50–59 age group (not represented in this study). Among all chronic HBV patients and HBsAg carriers in the region, the most represented groups were 30–39 (27%) and 40–49 year-olds (25%) [45]. Of particular concern is the positive correlation between anti-HBcore IgG prevalence and age, and its negative correlation with vaccination rates. This phenomenon likely has two explanations. First, like the age-related decline in anti-HBs IgG, it reflects the phased implementation of HBV vaccination in Russia, resulting in lower vaccination coverage among older age groups. Second, the statistical probability of exposure to infection increases with age. Notably, we found no correlation between HBsAg frequency and either age or vaccination rates.

Russian regional epidemiological studies report HBsAg detection rates of 1.5–3.5% among pregnant women [46,47], consistent with our results. In the study group, the frequency of HBsAg and anti-HBcore IgG was significantly higher among women with third or fourth pregnancies compared to those with first or second pregnancies, while the prevalence of anti-HBs IgG was significantly higher in the group with first/second pregnancies than in the group with third/fourth pregnancies. One might hypothesize that these differences are related to participant age, as younger women naturally have fewer pregnancies. We consider this assumption about age relevance valid for anti-HBs IgG occurrence, though it should be noted that this correlation is indirect. The true correlation, as mentioned earlier, is associated with vaccination and the national vaccination program implementation. However, it is noteworthy that while no differences in HBsAg frequency were found between age groups, HBsAg occurrence was significantly higher among women with third/fourth pregnancies compared to those with first/second pregnancies. The maximum frequency of anti-HBcore IgG in the oldest age group was 28.3%, while among women with third/fourth pregnancies (excluding the single case with five pregnancies) this frequency reached 43%.

No significant differences in HBV DNA frequency were detected between age groups in sequential pairwise comparisons. However, HBV DNA occurrence was significantly higher in the 32–36 age group compared to the 22–25 age group (OR = 2.6). At the same time, significant differences in HBV DNA frequency were found depending on the number of pregnancies (excluding the single case with five pregnancies). Sequential pairwise comparisons showed that HBV DNA detection frequency was significantly higher among women with two pregnancies compared to those with one pregnancy (OR = 2.04). Furthermore, the proportion of HBV DNA-positive individuals was higher among women with three pregnancies than among those with two pregnancies (OR = 3.4). Clearly, as with HBsAg, the increased HBV DNA frequency in these groups is associated more with the number of pregnancies than with age.

Risk factors for HBV infection include multiple medical interventions (including blood transfusions), intravenous drug use, having an HBV-infected sexual partner, lack of vaccination history, and residence in highly endemic regions [48]. The region where the pregnant women in our study reside is not highly endemic for HBV. During participant selection, we excluded individuals with a history of HIV infection, tuberculosis, parenteral viral hepatitis, past or present injection drug use, tattoos, or surgical interventions/blood transfusions unrelated to previous pregnancies. Given these inclusion/exclusion criteria, the increased frequencies of anti-HBcore IgG, HBsAg, and HBV DNA among women with more pregnancies are likely attributable to two primary factors: greater frequency of unprotected sexual contact; and medical interventions during previous deliveries. Overall, the observed increase in marker frequencies with age and number of pregnancies reflects the changing epidemiology of HBV in Russia, particularly the shift in transmission routes. Sexual transmission and parenteral medical procedures (as well as non-medical exposures like intravenous drug use and tattoos) now predominate over other modes of transmission [37,39]. The similarity between marker frequencies in our study and the general population indirectly supports the current predominance of heterosexual HBV transmission.

Our study revealed high OBI frequency (2.8%) in the cohort. HBV DNA prevalence exceeded population levels (0.4% for HBV DNA + HBsAg and 1.7% for OBI), showing similarity with St. Petersburg blood donors (0.4% for HBV DNA + HBsAg and 2.7% for OBI) [42]. This similarity likely reflects that ≈70% of donors in the reference study were young (18–40 years old), representing a demographic with high sexual activity.

Most vaccinated, HBV DNA-positive women were HBsAg-negative. While most pregnant women with OBI had concurrent anti-HBcore IgG antibodies, two women showed no serological markers, and one had only anti-HBs IgG. Standard testing would have missed these HBV infections, and only OBI testing accounting for low viral loads enabled their detection.

Of particular note are cases with concurrent detection of anti-HBs IgG and anti-HBc IgG (3.4%), regardless of HBV DNA status. Researchers from Novosibirsk reported similar prevalence (5.8%) of this antibody combination among HBsAg-negative pregnant women [49]. At least two factors may explain this phenomenon. First, HBV marker testing is not routinely performed prior to vaccination, meaning some vaccinated individuals might have been previously infected. Indirect support for this comes from the higher proportion of anti-HBc IgG among unvaccinated older individuals, and the fact that among pregnant women with anti-HBs IgG + anti-HBc IgG profile, over 85% were older than 26 years, compared to 11.1% aged 22–25 and 3.7% aged 18–21. Second, post-vaccination infection with immune escape HBV variants may occur. Both scenarios likely coexist in our study population.

In endemic regions, 8–12% of newborns from mothers with active CHB remain HBsAg-positive at one year despite immunization, highlighting the need to investigate prophylaxis failure causes [50]. 

While vertical transmission remains a significant concern for HBsAg-positive pregnant women with high viral loads, documented cases exist of virus transmission from HBsAg-negative mothers [51]. Cases have been reported of HBV transmission to newborns from mothers with undetectable plasma HBV DNA but detectable DNA in PBMCs [52]. Furthermore, although vertical transmission risk with OBI is relatively low, the potential for early horizontal transmission remains. This risk stems from two factors. First, HBV demonstrates remarkable environmental stability, surviving on surfaces at room temperature for up to seven days [53]. Second, the infectious dose is extremely low, just 3.5 IU/mL (approximately 16 viral copies) [8]. Thus, despite frequent asymptomatic HBV infection in pregnancy and typically low viral loads with OBI, the virus remains potentially dangerous for both mother and child.

The distribution of HBV genotypes in the study cohort warrants special attention. In Russia, the predominant genotypes D and A are more likely to cause chronic infection, with genotype A showing better response to interferon therapy, while genotype D responds poorly to such treatment. This represents a challenge compounded by its characteristic high mutation rate [18,54]. The predominance of genotype D with some presence of genotype A is typical for Russia overall and St. Petersburg specifically [55]. 

In our study, HBV subgenotypes among pregnant women were distributed as follows: D2—48.4%, D1—25.0%, D3—20.3%, A2—4.7%, and C1—1.5%. We previously described HBV genotypic profiles in various St. Petersburg populations. Among military personnel with chronic HBV, subgenotype analysis showed slightly elevated prevalence of D1 and D3, but still with significant D2 dominance: D2—58%, D1—20.9%, D3—16.3%, and A2—4.8% [56]. Individuals with newly diagnosed HIV and HBV coinfection (including occult infection) showed similar distribution: D2—55.6%, D1—22.2%, D3—13.9%, and A2—8.3% [57]. Surprisingly, blood donors exhibited equal proportions of D1 and D2 subgenotypes at 40.91% each [42]. However, comparative analysis revealed no significant differences in genotype distribution between pregnant women, blood donors, chronic HBV patients, and HIV/HBV-coinfected individuals.

The gradual decline in D2 prevalence in the region, from approximately 80% in previous studies to the current 40–60% across key populations, may reflect either sampling variations or true epidemiological changes due to increasing D1 and D3 prevalence in St. Petersburg, potentially driven by migration patterns from Central Asia [58].

The variability we identified in the genomic regions of genotype D HBV (the predominant genotype among the examined pregnant women) aligns with existing data about this variant’s naturally high variability. This characteristic is associated with disease chronicity and progression, as well as poor response to interferon-based therapy [54].

Genetic changes involving amino acid substitutions in the reverse transcriptase domain, when detected in treatment-naive individuals, are classified as either primary drug resistance mutations or secondary compensatory mutations that restore viral replication capacity. In this study, resistance mutations to lamivudine, entecavir, and telbivudine were identified in 4.69% of cases. All examined pregnant women denied receiving any treatment with these medications. We hypothesize these mutations in treatment-naive women either resulted from infection with pre-existing mutant virus or represent natural viral polymorphisms. The detection of polymorphic variant L80F (not previously documented as drug-resistant, but located at a position linked to lamivudine/telbivudine resistance) indirectly supports the latter hypothesis. Notably, while some evidence suggests lamivudine resistance mutations select for highly replicative HBV variants [24], in our cohort these mutations were found exclusively in women with OBI. This may indicate impending increases in viral replication activity, which could precipitate disease flares and elevate vertical transmission risk.

Of particular interest, all A2 isolates carried the L217R substitution in the RT region. Between 8 and 15% of patients initiating adefovir therapy show primary non-response, with several studies linking the natural L217R polymorphism (characteristic of genotype A2) to reduced adefovir susceptibility [59]. We additionally identified six cases of the M129L mutation in the reverse transcriptase region, a change some researchers associate with potential tenofovir resistance [60]. Among HBV DNA-positive pregnant women, 18.75% carried the M309K substitution, which some studies suggest confers a 3.5-fold higher HCC risk compared to wild-type HBV [61].

Both *in vivo* and *in vitro* studies have demonstrated that modifications in the MHR can alter the antigenic properties of HBsAg, reducing its immunogenicity and weakening the anti-HBs antibody response [62]. Among our findings, the near-universal presence of escape mutations appears particularly noteworthy. Polymorphic variants at three positions (T113S/P, T114S, N131T) were detected in all genotype D HBV samples. A substantial proportion of samples also exhibited substitutions at positions 118, 127, and 128 (46.9%, 39.1%, and 39.1%, respectively). Although some researchers have associated mutations at these positions with immune escape, the identified polymorphisms appear characteristic of genotype D and may either represent non-escape variants or indirectly suggest this genotype’s greater propensity for developing OBI. The predominant detection of mutations at positions 118, 127, and 128 in OBI cases further supports their potential role in OBI development with genotype D HBV. This hypothesis requiring additional investigation.

Anti-HBs IgG antibodies induced by HBV vaccination primarily target the hydrophilic “a” determinant region (amino acids 124–147) of the major HBsAg protein. Our analysis identified several well-characterized vaccine-escape mutations: P120T/L/S/Q, T126S/N, Q129R/H, M133I, D144G/E/H, and G145A/R/E, along with substitutions at known escape-associated positions (K141R, P142L) [63]. Furthermore, numerous S gene mutations detected in our cohort correlate with OBI development, including changes both within and outside the “a” determinant: Y100SY/C/F, Q101R/H, P120T/L/S/Q, C124R/W/G, T126S/N, Q129R/H, S143P/L/T, D144G/E/H, G145A/R/E, S167L/P, R169H, S174N, and V177A, plus novel OBI-associated mutations (G119R, R122K/I/Q, Y134H/N/F/D, C139W/F, K141R) [63,64,65]. It is also important to note that the variability of this region may affect the effectiveness of HBsAg detection kits [66], which, in turn, could have skewed the ratio between HBsAg+ and HBsAg− viral hepatitis B. Clarifying this information requires additional studies with an expanded range of kits. The clinical significance of these mutations is underscored by their detection in vaccinated, anti-HBs IgG-positive pregnant women with OBI. Such immune-associated escape mutations may interfere with vaccine-induced antibody recognition of HBsAg, posing a potential threat to global vaccination efforts [67]. Previous studies have reported HBsAg mutation frequencies ranging from 11% in North American populations to 47% among CHB patients in South Korea, with various MHR mutation rates (57.5% in genotype A, 100% in genotype D, 59.9% in genotype E) [68]. These findings are consistent with the high substitution rates we observe.

While expanded HBV vaccination has significantly reduced transmission risks, 5–10% of healthy vaccine recipients fail to mount an adequate immune response, a phenomenon confirmed in our study. Notably, many OBI cases feature concurrent viral DNA and vaccine-induced antibodies, with patients confirming prior vaccination [69]. Our results align with these observations. We hypothesize that the identified escape mutations contribute to the high OBI prevalence among pregnant women in our cohort as follows. By altering the protruding loop of the “a” determinant, these mutations prevent proper epitope recognition by pre-existing neutralizing antibodies, enabling mutant viruses to evade immune neutralization [17,70].

With the exception of rare deletion cases, most mutations in the precore/core region are point substitutions, primarily associated with reduced HBeAg levels and/or decreased viral load. Moreover, mutations in the core region are predominantly localized within immunologically active areas (MHC classes I + II) and may thus influence disease progression. For instance, mutations in the precore region (G1896A) and BCP (T1753C, A1762T/G1764A), as well as in the core domain (F24Y, E64D, E77Q, A80I/T/V, L116I, E180A), are known to correlate with severe liver disease and hepatocellular carcinoma (HCC) development [26]. 

In our study group, evaluation of clinically significant amino acid substitutions in the HBV genome revealed a high prevalence (18.8%) of the double mutation A1762T/G1764A in the BCP region. This mutation is strongly associated with liver cirrhosis and HCC [71,72,73].

Importantly, this mutation is associated with more frequent HCC progression independent of HBV viral load [74] and can be detected nearly a decade before HCC onset, positioning it as an early marker of hepatocarcinogenesis [75]. Interestingly, the presence of the BCP double mutation (A1762T/G1764A) in mothers was linked to the absence of vertical HBV transmission. This suggests a potential protective effect against perinatal viral spread, although an association with relatively low viremia titers in infected women cannot be ruled out [76]. Thus, in pregnant women with HBV infection and low viral load, the presence of the BCP A1762T/G1764A double mutation may indicate a reduced risk of pathogen transmission to the child, but warrants intensified monitoring of disease dynamics during and after pregnancy.

A significant number of mutations (39.1%) were detected in the precore region of HBV genomes from pregnant women, including several with established clinical significance. Particularly noteworthy was the high frequency of mutations at nucleotide position 1896 (15.63%), with the G1896A substitution (W28 stop codon) identified in 7.8% of cases. This mutation causes premature termination of the HBeAg precursor and is responsible for over 90% of defective HBeAg secretion cases, effectively abolishing antigen expression. The G1896A frequency in genotypes A and D may also increase with disease progression, as suggested by detected W28W and W28*Y variants [77].

The G29D (G1899A) mutation, found in 17.19% of our cases, similarly promotes disease progression, liver cirrhosis, and HCC [72,73]. Another clinically relevant precore substitution, H5D/V, is also associated with severe liver disease [72].

**Figure 6:**
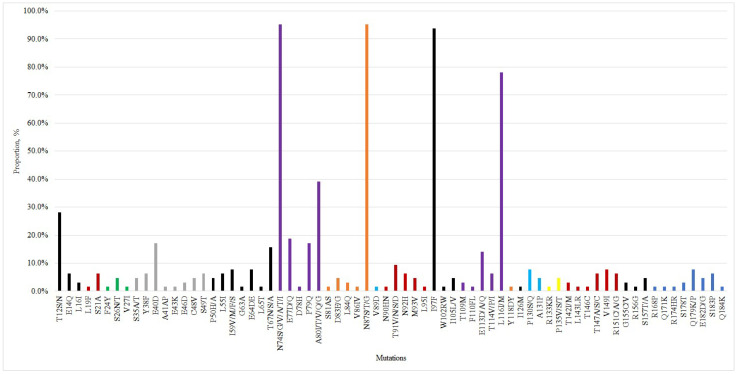
Localization of identified core region mutations within T-cell and B-cell epitope sites. The following are shown: black (group 1)—target epitopes for human CD4+ T cells; green (group 2)—cytotoxic T lymphocytes/CD8+ T cells; purple (group 3)—B-cell epitopes; red—groups 1 + 2; orange—groups 1 + 3; yellow—groups 2 + 3; blue—groups 1 + 2 + 3.

While most core region amino acid substitutions lack well-characterized clinical significance, several occur within known HBcAg immune recognition sites: human CD4+ T-cell epitopes (aa 1–20, 50–69, 81–105, 117–131, 141–165); cytotoxic CD8+ T-cell epitopes (aa 18–27, 88–96, 130–140, 141–151); and B-cell epitopes (aa 74–89, 107–118, 127–138). Mutations in these immunologically active regions critically influence viral persistence, host immune responses, and disease progression [72]. Thus, several identified substitutions, particularly those within T-cell and B-cell epitopes (Figure 6), may contribute to CHB development through immune evasion mechanisms.

Amino acid substitutions in key immune epitopes may potentially disrupt immune responses. This may contribute to the development of HBsAg-negative HBV, lead to persistent infection, or increase variability across all viral genomic regions [78].

In the study cohort, a significant number of mutations were identified in the core region (positions 113–143) that affect antigenicity and particle stability, giving rise to immune-escape mutants associated with chronic viral persistence [72,73]. For example, the rare mutations L143LR and T146C facilitate immune evasion, drive the selection of specific antibodies, promote chronic viral persistence, and accelerate disease progression, liver cirrhosis, and HCC. Additionally, these mutations enhance the formation of cccDNA during intracellular amplification while weakening infectivity [79]. Among the detected mutations, several are strongly linked to liver cirrhosis and HCC, including those in B-cell epitopes (E77D/Q, A80T, L116I/V/G) and T-cell epitopes (E64D, T91N/S). However, a recent study demonstrated that the presence of BCP/PC mutants in peripheral blood mononuclear cells of pregnant women did not lead to overt HBV infection in infants receiving full immunoprophylaxis, nor did it increase maternal liver disease risk over a 4-year follow-up period [80].

Notably, in our cohort of pregnant women, HBsAg-negative samples and those with low viral load exhibited greater variability across the precore/core region, possibly due to HBV replication inhibition by precore/core mutations.

While numerous studies have described an association between precore/core mutation frequency and liver disease progression in HBV-infected patients, the relationship between specific amino acid substitutions and clinical severity varies significantly across populations and even among studies of the same population. This discrepancy can be attributed to multiple factors [72], including viral genotype, patient ethnicity, host immune competence, and coinfection with other viruses. Nevertheless, certain mutations with established links to liver cirrhosis and HCC, as well as those affecting HBeAg serostatus, may serve as diagnostic and prognostic markers for early detection of liver disease progression in HBV-infected individuals.

## 4. Materials and Methods

### 4.1. Materials

**Figure 7:**
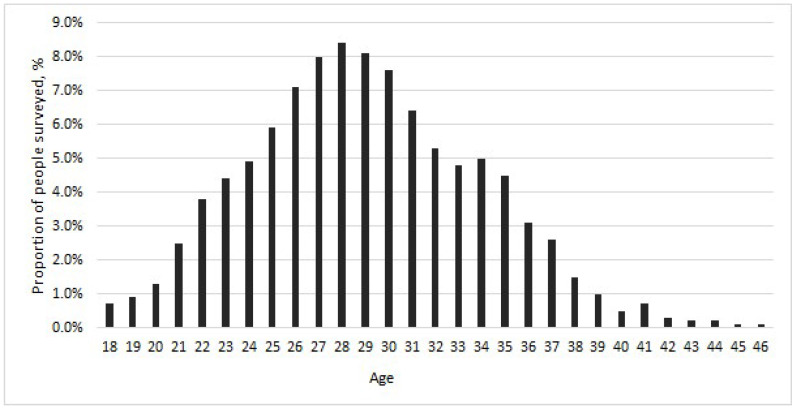
Age distribution of pregnant women in the study cohort.

The study material consisted of plasma samples obtained from 1368 pregnant women residing in St. Petersburg, Russia, collected during routine medical examinations at healthcare facilities. De-identified medical records provided information on age, chronic conditions, bloodborne infections, number of pregnancies, and HBV vaccination status. Exclusion criteria included a history of HIV infection, tuberculosis, parenteral viral hepatitis, past or present injection drug use, tattoos, as well as surgical procedures or blood transfusions unrelated to previous pregnancies. All participants were informed about the study (objectives, methodology) and provided written informed consent. The study was approved by the local ethics committee of the Saint Petersburg Pasteur Institute (protocol No. 151, dated 21 September 2021). Women’s ages ranged from 18 to 46 years, with a mean age of 29.06 years. The proportion of each age group among the participants was assessed (Figure 7).

For subsequent analysis, pregnant women were divided into several age groups based on their representation in the study population: Group 1 with less than 3% representation (ages 18–21); Group 2 with 3–5.9% representation (ages 22–25); Group 3 with 6–9% representation (ages 26–31); Group 4 with 3–5.9% representation (ages 32–36); and Group 5 with less than 3% representation (ages 37–46).

Among participants, the majority were experiencing their first pregnancy, namely 693 women (50.7%). Other histories included: 553 (40.4%) in their second pregnancy; 115 (8.4%) in their third pregnancy; 6 (0.4%) in their fourth pregnancy; and one woman (0.07%) in her fifth pregnancy.

### 4.2. Methods

#### 4.2.1. Sample Transportation and Storage

For serological and molecular biological testing of HIV and viral hepatitis markers, we used blood plasma collected from the antecubital vein into sterile disposable K_2_-EDTA anticoagulant tubes (10 mL volume) after overnight fasting. Plasma separation was achieved by centrifugation at +4 °C (1000× *g*, 3000 rpm) for 10 min. To prevent false-negative results in low viral load cases, all samples underwent preliminary virus concentration through ultracentrifugation (24,000× *g*, +4 °C, 1 h). Aliquots were distributed into cryovials for storage and subsequent testing: ELISA (500 μL); PCR (500 μL); and PCR with sequencing (5000 μL). Samples were transported within 24 h of collection using specialized biomaterial containers maintained at +4 to +8 °C.

#### 4.2.2. Enzyme-Linked Immunosorbent Assay

ELISA analysis for HBV markers involved qualitative determination of HBsAg, anti-HBs IgG, and anti-HBcore IgG as previously described [12]. Tests were repeated twice using reagents in accordance with manufacturer instructions: DS-EIA-HBsAg, DS-EIA-ANTI-HBsAg, and DS-EIA-ANTI-HBc (Diagnostic Systems RPC, Nizhny Novgorod, Russia); and Vectohep B-HBs-antigen, VectoHBsAg-antibodies, and HepaBest anti-HBc-IgG commercial kits (Vector-Best, Novosibirsk, Russia).

#### 4.2.3. Nucleic Acid Extraction

DNA extraction from 500 μL of plasma was performed for PCR screening. HBV DNA-positive samples underwent additional extraction from ≥5 mL plasma to obtain sufficient material for sequencing using the “NK-Magno-UltraPure-A” reagent kit (LLC “NPF Epitop”, Saint Petersburg, Russia) per manufacturer’s instructions with modifications to increase the volume of material.

#### 4.2.4. Polymerase Chain Reaction and Sequencing

Qualitative HBV DNA detection was performed using an in-house method developed at the Saint Petersburg Pasteur Institute, capable of identifying the pathogen at viral loads ≥ 5 IU/mL, including HBsAg-negative chronic HBV cases [81,82]. As recommended by Taormina Workshop on Occult HBV Infection faculty members, when the virus was detected, nested PCR was applied using a set of primers co-flanking the full viral genome, as described previously [9,11,12,83].

#### 4.2.5. Genotype and Mutation Analysis

Consensus sequence assembly from sequencing fragments was performed using “Unipro UGENE” v. 47 software [84]. Primary analysis of the obtained fragments was performed using the Nucleotide BLAST algorithm (https://blast.ncbi.nlm.nih.gov/Blast.cgi (accessed on 1 May 2025)) on the nucleotide sequences provided in the GenBank sequence database. The resulting sequences were submitted to the GenBank sequence database. The resulting sequences were aligned in the MEGAv.11 program using the ClustalW algorithm [85]. The phylogenetic tree was constructed using the neighbor-joining method. The significance of the tree was assessed using bootstrap analysis with 1000 replicates. The nucleotide sequences obtained were submitted to the HBVseq (https://hivdb.stanford.edu/HBV/HBVseq/development/HBVseq.html (accessed on 5 May 2025)) [86], HBVdb (https://hbvdb.lyon.inserm.fr/HBVdb/HBVdbIndex (accessed on 5 May 2025)) [87], and Genafor (https://hbv.geno2pheno.org (accessed on 5 May 2025)) [88] databases to search for possible mutations. Protein amino acid sequences were determined by translating the corresponding nucleotide sequence according to the open reading frame.

#### 4.2.6. Statistical Analysis

Statistical data processing was carried out using the Excel (Microsoft Corp., Redmond, WA, USA) and Prizm 5.0 (GraphPad Software, Inc., San Diego, CA, USA; https://www.graphpad.com/support/prism-5-updates/ (accessed on 5 May 2025)) [89] software packages. To assess statistical error, the exact Cloepper-Pearson interval was applied. Results are presented with a 95% confidence interval (CI). For assessing the significance of differences in quantitative data during paired comparisons, either Fisher’s exact test or the Chi-square test with Yates’ correction was used depending on the characteristics of the samples. A probability value of *p* < 0.05 was set as the threshold for statistical significance. Correlation analysis was conducted, taking into account compliance with parametric distribution, with calculation of Spearman rank correlation coefficients (rs). Differences were considered statistically significant when *p* < 0.05.

## 5. Conclusions

Despite widespread hepatitis B vaccination programs, the issue of revaccination remains relevant both for high-risk groups and the general population due to the gradual waning of vaccine-induced immunity over time. Monitoring collective immunity levels and improving hepatitis B diagnostics are crucial for optimizing epidemiological surveillance, implementing targeted prevention measures, and predicting the effectiveness of prophylactic strategies.

Our findings reveal a high prevalence of OBI among pregnant women in St. Petersburg. The detection of HBsAg-negative hepatitis B reflects circulating viral variants carrying escape mutations that both evade HBsAg recognition by diagnostic antibodies and enable the virus to circumvent vaccine-induced immunity. Concurrent mutations may cause diagnostic test failures (i.e., with HBsAg detection), and they have the potential to reduce prophylactic efficacy of various types (immunoglobulins, vaccines), triggering viral reactivation or disease progression. The predominance of HBV isolates in pregnant women harboring concurrent mutations clearly poses a significant public health threat that warrants further investigation.

## Figures and Tables

**Table 1:** Proportion of HBV-vaccinated individuals across different age groups in the study population.

Age Group, Years	Number of Volunteers	Vaccinated Against HBV	
	N	abs, (n)	%, 95% CI
18–21 years	74	67	90.5% (81.5–96.1%)
22–25 years	260	231	88.9% (84.4–92.4%)
26–31 years	624	533	85.4% (82.4–88.1%)
32–36 years	311	206	66.2% (60.7–71.5%)
37–46 years	99	51	51.5% (41.3–61.7%)
Total:	1368	1088	79.5% (77.3–81.6%)

**Table 2:** Prevalence of serological markers among pregnant women in different age groups.

Age Group, Years	Number of Volunteers	Anti-HBs IgG		Anti-HBcore IgG		HBsAg		Anti-HBs IgG + Anti-HBcore IgG	
	N	abs, (n)	%, 95% CI	abs, (n)	%, 95% CI	abs, (n)	%, 95% CI	abs, (n)	%, 95% CI
18–21 years	74	56	75.7% (64.3–84.9%)	4	5.4% (1.5–13.3%)	0	0% (0–4.9%)	2	0.15% (0.02–0.53%)
22–25 years	260	181	69.6% (63.6–75.2%)	16	6.2% (3.6–9.8%)	2	0.8% (0.1–2.8%)	6	0.44% (0.16–0.95%)
26–31 years	624	419	67.2% (63.3–70.8%)	58	9.3% (7.1–11.9%)	11	1.8% (0.9–3.1%)	18	1.32% (0.78–2.07%)
32–36 years	311	169	54.3% (48.6–60.0%)	71	22.8% (18.3–27.9%)	10	3.2% (1.6–5.8%)	18	1.32% (0.78–2.07%)
37–46 years	99	48	48.5% (38.3–58.8%)	28	28.3% (19.7–38.2%)	3	3.0% (0.6–8.6%)	10	0.73% (0.35–1.34%)
Total:	1368	873	63.8% (61.2–66.4%)	177	12.9% (11.2–14.8%)	26	1.9% (1.3–2.8%)	54	3.95% (2.98–5.12%)

**Table 3:** Prevalence of serological markers among study participants by pregnancy number.

Pregnancy Number	Number of Volunteers	Anti-HBs IgG		Anti-HBcore IgG		HBsAg	
	N	abs, (n)	%, 95% CI	abs, (n)	%, 95% CI	abs, (n)	%, 95% CI
5th pregnancy	1	0	0.0% (0.0–97.5%)	1	100.0% (2.5–100%)	0	0.0% (0.0–97.5%)
4th pregnancy	6	2	33.3% (4.3–77.7%)	4	66.7% (22.3–95.7%)	1	16.7% (0.4–64.1%)
3rd pregnancy	115	62	53.9% (44.4–63.3%)	49	42.6% (33.4–52.2%)	7	6.1% (2.5–12.1%)
2nd pregnancy	553	357	64.6% (60.4–68.6%)	65	11.8% (9.2–14.8%)	11	2.0% (1.0–3.5%)
1st pregnancy	693	452	65.2% (61.6–68.8%)	58	8.4% (6.4–10.7%)	7	1.0% (0.4–2.1%)

**Table 4:** Distribution of serological markers among pregnant women.

Hepatitis B Serological Markers	Prevalence	
	abs, (n), N = 1368	%, 95% CI
HBsAg	9	0.7% (0.3–1.3%)
Anti-HBs IgG	818	59.8% (57.1–62.4%)
Anti-HBc IgG	115	8.4% (7.0–10.0%)
HBsAg, anti-HBs IgG	1	0.1% (0.0–0.4%)
HBsAg, anti-HBc IgG	8	0.6% (0.3–1.2%)
HBsAg, anti-HBs IgG, anti-HBc IgG	8	0.6% (0.3–1.2%)
Anti-HBs IgG, anti-HBc IgG	46	3.4% (2.5–4.5%)

**Table 5:** Distribution of serological markers in the study group by number of pregnancies.

Hepatitis B Serological Markers	Number of Volunteers	1st Pregnancy		2nd Pregnancy		3rd Pregnancy		4th Pregnancy		5th Pregnancy	
	N	abs, (n)	%, 95% CI	abs, (n)	%, 95% CI	abs, (n)	%, 95% CI	abs, (n)	%, 95% CI	abs, (n)	%, 95% CI
HBsAg	9	2	22.2% (2.8–60.0%)	4	44.4% (13.7–78.8%)	2	22.2% (2.8–60.0%)	1	11.1% (0.3–48.3%)	0	0.0% (0.0–33.6%)
Anti-HBs IgG	818	433	52.9% (49.5–56.4%)	336	41.1% (37.7–44.5%)	48	5.9% (4.4–7.7%)	1	0.1% (0.0–0.7%)	0	0.0% (0.0–0.5%)
Anti-HBc IgG	115	39	33.9% (25.4–43.3%)	41	35.7% (26.9–45.1%)	31	26.9% (19.1–36.0%)	3	2.6% (0.5–7.4%)	1	0.9% (0.0–4.8%)
HBsAg, anti-HBs IgG	1	1	100.0% (2.5–100%)	0	0.0% (0–97.5%)	0	0.0% (0.0–97.5%)	0	0.0% (0.0–97.5%)	0	0.0% (0.0–97.5%)
HBsAg, anti-HBc IgG	8	1	12.5% (0.3–52.7%)	3	37.5% (8.5–75.5%)	4	50.0% (15.7–84.3%)	0	0.0% (0.0–36.9%)	0	0.0% (0.0–36.9%)
HBsAg, anti-HBs IgG, anti-HBc IgG	8	3	37.5% (8.5–75.5%)	4	50.0% (15.7–84.3%)	1	12.5% (0.3–52.7%)	0	0.0% (0.0–36.9%)	0	0.0% (0.0–36.9%)
Anti-HBs IgG, anti-HBc IgG	46	15	32.6% (19.5–48.0%)	17	36.9% (23.2–52.5%)	13	28.3% (16.0–43.5%)	1	2.2% (0.1–11.5%)	0	0.0% (0.0–7.7%)

**Table 6:** Prevalence of HBV DNA among pregnant women by age group.

Age Group, Years	Number of Volunteers	HBV DNA	
	N	abs, (n)	%, 95% CI
18–21 years	74	1	1.35% (0.0–7.3%)
22–25 years	260	7	2.7% (1.1–5.5%)
26–31 years	624	30	4.8% (3.3–6.8%)
32–36 years	311	21	6.8% (4.2–10.1%)
37–46 years	99	5	5.1% (1.7–11.4%)
Total:	1368	64	4.7% (3.6–5.9%)

**Table 7:** Prevalence of HBV DNA in the study group by pregnancy number.

Pregnancy Number	Number of Volunteers	HBV DNA	
	N	abs, (n)	%, 95% CI
5th pregnancy	1	0	0.0% (0.0–97.5%)
4th pregnancy	6	3	50.0% (11.8–88.2%)
3rd pregnancy	115	17	14.8% (8.9–22.6%)
2nd pregnancy	553	27	4.9% (3.2–7.0%)
1st pregnancy	693	17	2.5% (1.4–3.9%)

**Table 8:** Prevalence of HBV DNA among pregnant women in different age groups by HBsAg status.

Age Group, Years	Number of Volunteers	HBV DNA+, HBsAg+		HBV DNA+, HbsAg−	
	N	abs, (n)	%, 95% CI	abs, (n)	%, 95% CI
18–21 years	74	0	0.0% (0.0–4.9%)	1	1.35% (0.0–7.3%)
22–25 years	260	2	0.8% (0.1–2.8%)	5	1.9% (0.6–4.4%)
26–31 years	624	11	1.8% (0.9–3.1%)	19	3.0% (1.8–4.7%)
32–36 years	311	10	3.2% (1.6–5.8%)	11	3.5% (1.8–6.2%)
37–46 years	99	3	3.0% (0.6–8.6%)	2	2.0% (0.3–7.1%)
Total:	1368	26	1.9% (1.3–2.8%)	38	2.8% (2.0–3.8%)

**Table 9:** Distribution of hepatitis B serological markers among HBV DNA-positive pregnant women.

Hepatitis B Serological Markers	HBV DNA Positive (N = 64)		HBV DNA+, HBsAg+ (N = 26)		HBV DNA+, HbsAg− (N = 38)	
	abs, (n)	%, 95% CI	abs, (n)	%, 95% CI	abs, (n)	%, 95% CI
HBsAg	9	14.1% (6.6–25.0%)	9	34.6% (17.2–55.7%)	0	0.0% (0.0–9.3%)
Anti-HBs IgG	1	1.6% (0.0–8.4%)	0	0.0% (0.0–13.2%)	1	2.6% (0.1–13.8%)
Anti-HBc IgG	25	39.1% (27.1–52.1%)	0	0.0% (0.0–13.2%)	25	65.8% (48.7–80.4%)
HBsAg, anti-HBs IgG	1	1.6% (0.0–8.4%)	1	3.9% (0.1–19.6%)	0	0.0% (0.0–9.3%)
HBsAg, anti-HBc IgG	8	12.5% (5.6–23.2%)	8	30.8% (14.3–51.8%)	0	0.0% (0.0–9.3%)
HBsAg, anti-HBs IgG, anti-HBc IgG	8	12.5% (5.6–23.2%)	8	30.8% (14.3–51.8%)	0	0.0% (0.0–9.3%)
Anti-HBs IgG, anti-HBc IgG	10	15.6% (7.8–26.9%)	0	0.0% (0.0–13.2%)	10	26.3% (13.4–43.1%)
Seronegative	2	3.1% (0.4–10.8%)	0	0.0% (0.0–13.2%)	2	5.3% (0.6–17.8%)

**Table 10:** Proportions of individuals vaccinated and unvaccinated against HBV among HBV DNA-positive pregnant women.

Vaccination Status	HBV DNA+, HBsAg+ (N = 26)		HBV DNA+, HbsAg− (N = 38)	
	abs, (n)	%, 95% CI	abs, (n)	%, 95% CI
vaccinated	1	3.85% (0.1–19.6%)	26	68.4% (51.4–82.5%)
unvaccinated	25	96.15% (80.4–99.9%)	12	31.6% (17.5–48.7%)

**Table 11:** Mutations identified in the MHR (aa 99–169) in the study group.

Mutation	Frequency of Occurrence in the Group (N = 64)			
	abs, n	%	95% CI −	95% CI +
Y100SY/C/F	4	6.3%	1.7%	15.2%
Q101R/H	2	3.1%	0.4%	10.8%
L109R	2	3.1%	0.4%	10.8%
I110S/L	4	6.3%	1.7%	15.2%
P111Q	2	3.1%	0.4%	10.8%
T113S/P	63	98.4%	91.6%	100.0%
T114S	62	96.9%	89.2%	99.6%
T118L/V/A/G/M	30	46.9%	34.3%	59.8%
G119E	1	1.6%	0.0%	8.4%
P120T/L/S/Q	5	7.8%	2.6%	17.3%
R122K/I/Q	6	9.4%	3.5%	19.3%
T123A	1	1.6%	0.0%	8.4%
C124R/W/G	3	4.7%	1.0%	13.1%
T125M	4	6.3%	1.7%	15.2%
T126S/N	2	3.1%	0.4%	10.8%
T127P	25	39.1%	27.1%	52.1%
A128V/A/G/F	25	39.1%	27.1%	52.1%
Q129R/H	4	6.3%	1.7%	15.2%
G130R/A	2	3.1%	0.4%	10.8%
N131T	61	95.3%	86.9%	99.0%
S132SY	1	1.6%	0.0%	8.4%
M133I	1	1.6%	0.0%	8.4%
Y134H/N/F/D	6	9.4%	3.5%	19.3%
C137Y/W	3	4.7%	1.0%	13.1%
C139W/F	2	3.1%	0.4%	10.8%
T140ST	1	1.6%	0.0%	8.4%
K141R	1	1.6%	0.0%	8.4%
P142L	1	1.6%	0.0%	8.4%
S143P/L/T	8	12.5%	5.6%	23.2%
D144G/E/H	5	7.8%	2.6%	17.3%
G145A/R/E	6	9.4%	3.5%	19.3%
N146S	1	1.6%	0.0%	8.4%
G159A/G/E	6	9.4%	3.5%	19.3%
E164G/K	5	7.8%	2.6%	17.3%
A166V	1	1.6%	0.0%	8.4%
S167L/P	3	4.7%	1.0%	13.1%

**Table 12:** Distribution of major escape mutations among pregnant women with CHB and OBI.

Mutation	Frequency of Occurrence in the CHB Group (N = 26)		Frequency of Occurrence in the OBI Group (N = 38)	
	abs, n	%, 95% CI	abs, n	%, 95% CI
P120T/L/S/Q	0	0%	5	13.2% (4.4–28.1%)
R122K/I/Q	1	3.85% (0.1–19.6%)	5	13.2% (4.4–28.1%)
C124R/W/G	0	0%	3	7.9% (1.7–21.4%)
T126S/N	0	0%	2	5.3% (0.6–17.7%)
Q129R/H	0	0%	4	10.5% (2.9–24.8%)
M133I	0	0%	1	2.6% (0.1–13.8%)
C137Y/W	0	0%	3	7.9% (1.7–21.4%)
Y134H/N/F/D	1	3.85% (0.1–19.6%)	3	7.9% (1.7–21.4%)
K141R	0	0%	1	2.6% (0.1–13.8%)
P142L	0	0%	1	2.6% (0.1–13.8%)
S143P/L/T	2	7.69% (0.9–25.1%)	6	15.8% (6.0–31.3%)
D144G/E/H	0	0%	4	10.5% (2.9–24.8%)
G145A/R/E	0	0%	5	13.2% (4.4–28.1%)

**Table 13:** S region mutations identified in HBV-vaccinated, anti-HBs IgG-positive individuals with OBI.

Sample	Vaccinated Against HBV	Anti-HBs IgG	Mutation
ocHBV_pw315	Yes	Yes	T113P, T114A, T118V, K122R, P127T, A128V, N131T, F134Y, G145R, A159G, Y161F, V168A
ocHBV_pw560	Yes	Yes	T113P, T114A, K122R, T126N, T127P, N131T, F134Y, D144E, A159G, Y161F, V168A
ocHBV_pw570	Yes	Yes	Y100C, I110L, T113P, T114A, T118G, K122R, P127T, A128V, N131T, F134Y, A159G, K160R, Y161F, W163S, E164K, W165C, V168A, V177A
ocHBV_pw770	Yes	Yes	D99A, L109R, T113S, T114S, T118V, K122R, P127T, A128V, N131T, F134Y, A159G, Y161F, E164G, S167L, V168A
ocHBV_pw863	Yes	Yes	T113S, T114S, K122R, T127P, N131T, F134Y, G145A, A159G, Y161F, V168A

**Table 14:** Mutations identified in the precore region in the study group.

Mutation	Frequency of Occurrence in the Group (N = 64)	
	abs, n	%, 95% CI
L3R	1	1.56% (0.04–8.40%)
H5D/V	2	3.13% (0.38–10.84%)
C12F	5	7.81% (2.59–17.30%)
C14G/S	7	10.94% (4.51–21.25%)
P15A	2	3.13% (0.38–10.84%)
A19G	5	7.81% (2.59–17.30%)
L22M	2	3.13% (0.38–10.84%)
W28*/*W/*Y	10	15.63% (7.76–26.86%)
G29D	11	17.19% (8.90–28.68%)

**Table 15:** Mutations identified in the core region in the study group.

Mutation	Frequency of Occurrence in the Group (N = 64)			
	abs, n	%	95% CI −	95% CI +
T12S/N	18	28.1%	17.6%	40.8%
E14Q	4	6.3%	1.7%	15.2%
L16I	2	3.1%	0.4%	10.8%
L19F	1	1.6%	0.0%	8.4%
S21A	4	6.3%	1.7%	15.2%
F24Y	1	1.6%	0.0%	8.4%
S26N/T	3	4.7%	1.0%	13.1%
V27I	1	1.6%	0.0%	8.4%
S35A/T	3	4.7%	1.0%	13.1%
Y38F	4	6.3%	1.7%	15.2%
E40D	11	17.2%	8.9%	28.7%
A41AP	1	1.6%	0.0%	8.4%
E43K	1	1.6%	0.0%	8.4%
E46D	2	3.1%	0.4%	10.8%
C48V	3	4.7%	1.0%	13.1%
S49T	4	6.3%	1.7%	15.2%
P50H/A	3	4.7%	1.0%	13.1%
L55I	4	6.3%	1.7%	15.2%
I59V/M/F/S	5	7.8%	2.6%	17.3%
G63A	1	1.6%	0.0%	8.4%
E64DE	5	7.8%	2.6%	17.3%
L65T	1	1.6%	0.0%	8.4%
T67N/S/A	10	15.6%	7.8%	26.9%
N74S/G/V/A/T/I	61	95.3%	86.9%	99.0%
E77D/Q	12	18.8%	10.1%	30.5%
D78H	1	1.6%	0.0%	8.4%
P79Q	11	17.2%	8.9%	28.7%
A80I/T/V/Q/G	25	39.1%	27.1%	52.1%
S81AS	1	1.6%	0.0%	8.4%
D83E/G	3	4.7%	1.0%	13.1%
L84Q	2	3.1%	0.4%	10.8%
V86IV	1	1.6%	0.0%	8.4%
N87S/T/G	61	95.3%	86.9%	99.0%
V89D	1	1.6%	0.0%	8.4%
N90HN	1	1.6%	0.0%	8.4%
T91V/N/S/D	6	9.4%	3.5%	19.3%
N92H	4	6.3%	1.7%	15.2%
M93V	3	4.7%	1.0%	13.1%
L95I	1	1.6%	0.0%	8.4%
I97F	60	93.8%	84.8%	98.3%
W102RW	1	1.6%	0.0%	8.4%
I105L/V	3	4.7%	1.0%	13.1%
T109M	2	3.1%	0.4%	10.8%
F110FL	1	1.6%	0.0%	8.4%
E113D/A/Q	9	14.1%	6.6%	25.0%
T114V/P/I	4	6.3%	1.7%	15.2%
L116I/M	50	78.1%	66.0%	87.5%
Y118DY	1	1.6%	0.0%	8.4%
I126M	1	1.6%	0.0%	8.4%
P130S/Q	5	7.8%	2.6%	17.3%
A131P	3	4.7%	1.0%	13.1%
R133KR	1	1.6%	0.0%	8.4%
P135V/S/T	3	4.7%	1.0%	13.1%
T142I/M	2	3.1%	0.4%	10.8%
L143LR	1	1.6%	0.0%	8.4%
T146C	1	1.6%	0.0%	8.4%
T147A/S/C	4	6.3%	1.7%	15.2%
V149I	5	7.8%	2.6%	17.3%
R151C/A/G	4	6.3%	1.7%	15.2%
G155C/V	2	3.1%	2.6%	4.2%
R156G	1	1.6%	1.1%	2.6%
S157T/A	3	4.7%	4.2%	5.7%
R168P	1	1.6%	1.1%	2.6%
Q171K	1	1.6%	1.1%	2.6%
R174HR	1	1.6%	1.1%	2.6%
S178T	2	3.1%	2.6%	4.2%
Q179K/P	5	7.8%	7.3%	8.8%
E182D/G	3	4.7%	4.2%	5.7%
S183P	4	6.3%	5.7%	7.3%
Q184K	1	1.6%	1.1%	2.6%

## Data Availability

The raw data supporting the conclusions of this article will be made available by the authors on request.
